# Bibliographical Notices, &c.

**Published:** 1846-01

**Authors:** 


A PRACTICAL TREATISE ON HEALTHY SKIN, WITH RULES FOR THE MEDICAL AND DOMESTIC TREATMENT OF CUTANEOUS DISEASES. By *Erasmus Wilson*, F.R.S. 8vo. pp. 356. London, 1845.

WE have perused this work with regret, and feel constrained to speak of it with disapproval, which we can the more readily do, inasmuch as our notices of Mr. Wilson's former publications prove that we have at all times been able to appreciate and do justice to their able and highly useful characteristics. It does not, however, follow that an author, who is well occupied in imparting to his professional brethren the results of his experience, research, or meditation, is properly employed when he comes to discourse upon similar matters with the public at large. There is in fact but one subject upon which a medical writer should ever allow himself to address the public directly upon—the all-important one of *Hygiene*, or the preservation of health and prevention of disease. Upon this he cannot be too communicative; and the numerous and valuable works, which have appeared relating to it, testify to the disinterestedness of our profession, as well as to its utility in other respects, besides being that of the art of healing. But it unfortunately happens that few authors, except perhaps Combe, have been contented to confine themselves within these limits, spacious as they are, but seem to consider it incumbent upon them to enter into anatomical and physiological details to an extent that can neither be comprehended or retained by their readers, or would prove beneficial to them if this were possible. Far worse than this, is the practice, become so general, of supplying popular descriptions of diseases, and of the means of treatment by which their removal may be most readily effected. Mr. Wilson's book is an example of both of the errors we have indicated; and exhibits a yet farther mischievous tendency in its advocacy of the hydropathic treatment of disease. All this might be expected, although not excused, at the hands of a man writing for popularity and notoriety; but is indeed grievous, when found to proceed from one who has acquired for himself a respectable professional *status*. So pernicious do we consider an example of this kind, that we must crave permission to say a few more words upon each of the points to which we have alluded.

First, then, the work treats too minutely of the anatomy of the skin. We are no opponents to the diffusion of knowledge, but we are quite at a loss to conceive the advantage that can ensue from the popularizing the results of microscopical investigations in anatomy. The general outlines of the structure and functions of the human frame may be advantageously communicated to the public, but all beyond this should be looked upon as professional knowledge, whose general diffusion would be useless and ridiculous. It is difficult enough for the man in practice to keep himself acquainted with the progress and even the nomenclature of medical and accessory science, albeit it is necessary for his improvement that he should take some pains so to do; and yet it is attempted to impart such knowledge to the mere *dilettante*, unprovided with any preliminary information whatever. Mr. Wilson, not content with presenting an epitome of the cell theory and plates and descriptions of the microscopical structure of the skin, feels it incumbent upon him to give a popular account of his paper on the *Entozoon Folliculorum* in the Philosophical Transactions. We look upon this as positively derogatory. Let a man record his investigations in appropriate channels for communication with those who can appreciate and understand, contradict or confirm, them; but surely he should avoid dragging them into public view for the mere purpose of exciting and gratifying the gaping wonderment of ignorant readers. We venture to say that Simon, the discoverer of the parasite, whose paper in Müller's Archiv. he refers to, would not have condescended to such showman-like proceedings. The taste, too, with which our author introduces to the notice of his lady-readers a magnified view and description of the Itch-animalcule, seems somewhat questionable.

Secondly, although some of our readers may not agree with us in considering the development of minute anatomical or scientific facts to the public, if unprofessional, or at all events, a very unworthy occupation for a professional man of any standing—yet we suppose that there are none but will join with us in deprecating the publication of popular accounts of the characters and treatment of disease. Well, here we have the author's classification of cutaneous affections stated, and superficial descriptions of each malady, and the means of managing it supplied. Mr. Wilson may tell us that he is ever and anon pointing out to his readers, that, when a disease is constitutional, serious, or obstinate, they must cease to rely upon their own judgment, and resort to professional aid. This is sheer nonsense. To enumerate a number of diseases, some of which are simple, others serious, and many of which may be readily confounded with each other; and to encourage people to believe that they can distinguish the one from the other, and even up to a certain point treat them, is equally unjust to the public as to the profession; holding out to the former hopes which can seldom be realized, and the attempting at whose fulfilment may often be attended with danger and delay, and depriving the latter of the opportunity of seeing diseases when they can be most effectually and most creditably treated, before bungling attempts at self-management have rendered them complicated and obstinate. The uninformed person, even as to the nature of his disease, will try first one plan and then another, with all the impatience and indiscrimination that ignorance engenders, and after exhausting all the author's precepts and formulæ (some of which contain substances ill-adapted for popular use), and finding his malady becoming worse instead of better, will at last be driven to resort to persons who have made the study of the detection and treatment of disease the business of their lives, and whose advice and assistance, but for the misleading of this book, he might have so much more advantageously sought in the first instance. So impossible does it seem to us for well-informed medical men to believe that they can advantageously instruct the public upon professional topics, that we always suspect their attempting to do so. A name and practice, it is true, may be so built up, and the inditing a book has frequently been the formula recommended for constructing one; but we look upon the addressing the public directly upon such topics as an illegitimate practice, and that the only sound procedure is to confine the exhibition of our acquirements to our professional brethren, who alone can appreciate them, and who are always willing to assist in making them lucratively known.

Thirdly.—The course Mr. Wilson has taken in respect to Hydropathy, is a very extraordinary one. He seems to have seen little or nothing of its practical operation, but furnishes large extracts from Dr. Wilson's work upon the subject, and from these has composed a glowing eulogium on the practice and its founder, Preisnitz. He is of course quite at liberty to entertain any opinion he pleases of the therapeutic powers of this remedy; but, even allowing he believes it may one day become, in the hands of medical practitioners, a powerful agent for the production of effects which are not producible in a less objectionable manner, which is indeed very much more than we should like to confess anticipating, even supposing he does or is about to try its efficacy among his patients, is he, when he knows it is at least *sub judice*, and its practical carrying out is, for the most part, *now* in the hands of quacks and impostors, justified in delivering, in a work like the present, so decided an opinion in its favour to the public, who will quote his authority and adopt his recommendation, irrespective of the very few cautionary observations by which it is accompanied. Moreover, there was no call for any notice of the system whatever, as his remarks are not directed to its application for the treatment of cutaneous affections in particular, but of disease in general. One caution he gives, some we have no doubt will take care to prominently bring under extensive public notice, viz. that it is only in hydropathic "institutions" that the water-cure can really be effectually carried out.* "I trust the day is not far distant when we shall see such institutions, *hygienic sanatoriums* (!) in fact, in the neighbourhood of all our large cities, and at our watering-places." We trust that the day is not far distant when men who have helped on the progress of medical knowledge, who are teachers of our rising generation of practitioners, and members of our only scientific society, men such as Herbert Mayo and Erasmus Wilson, will cease to ally themselves with and defend the gross but popular delusions of their epoch. We have thus not only to complain of the assaults of quackery from without, but every now and then to endure the mortification of witnessing honoured members of our own body quit our ranks, place themselves side by side with quacks, impostors, and amateur doctors, and do battle against us.

We do not deny that Mr. Wilson's volume contains some valuable observations relative to hygiene, although this subject has been far more ably and amply dealt with by Combe. Extricated from the miscellaneous information on minute ana-

---

* It may gratify our author to learn, that no less a personage than Sir Edward Bulwer Lytton is of the same opinion with himself upon this point. Not satisfied with the honours of the poet and ready novel-writer, the literary Baronet has become an *amateur-medecin.* In his "Confessions of a Water Patient"—a pretty little book for the drawing-room table—he records the results not only of his own personal experience, but also of his observations upon others, in various places, and especially at "beloved Malvern." As a matter of course, the narrative is adorned with classic allusions, scraps of poetic thought, and sweet sentimentalisms. The wet sheet is described as "a magic girdle in which pain is lulled, and fever cooled, and watchfulness lapped in slumber;" and then there is something about "Undine in her virgin existence and in her wedded state," which we must confess that we do not understand. There is one exquisite *morçeau,* which we must find room for. After a glowing description of the delights of a hydropathic establishment, the Baronet triumphantly exclaims;—"Compare this life, O merchant, O trader, O man of business, escaping to the sea-shore, with that which you there lead—with your shrimps and your shell fish, and your wine and your brown stout—with all which counteracts, in the evening, the good of your morning dip and your noonday stroll."

tomy, receipts for preserving the complexion, instructions for washing the face and paring the nails, eulogia on hydropathy, descriptions of diseases and formulæ for treatment, they would form but a thin pamphlet which might even be spared. Notwithstanding what we have said of the work, we do not doubt (for its faults will prove with many its attractions) it will soon reach a second edition; and we would suggest to its author, as a means of giving additional attraction to it in the eyes of the classes who will be its chief perusers, the furnishing, in an appendix, a succint account of the *Syphilides*—a class of diseases in which hydropathy is vaunted as pre-eminently adapted for treating!

---

### A Memoir on Amputation of the Thigh at the Hip-joint (with a successful Case.) By *William Sands Cox*, F.R.S,, &c.

Mr. Cox, having performed a successful operation at the hip-joint, ventures, as he says, *with diffidence, to call the attention of the profession* to the subject, and to the case herein recorded, under the firm conviction that very many lives may be saved by the operation. We have an equally firm conviction that, in civil life, the cases in which such an operation is required are extremely rare, and that, if the operation were to be often performed, very many lives would be sacrificed instead of being saved; and we know that such is the opinion of the best surgeons in the present day. Mr. Cox, after briefly alluding to the numerous successful and unsuccessful cases of amputation at the hip-joint, proceeds to enumerate the causes which may render the operation justifiable. These he gives as follows:—1st. Compound fractures and compound dislocations, or other severe injuries, of the head, neck, and upper part of the femur. 2dly. The removal of the thigh by machinery. 3rdly. Mortification. 4th. Caries and osteonecrosis. 5th. Exostosis and Hyperostosis. 6th. Various tumors of a nonmalignant character, viz. osteo-sarcoma, osteo-steatoma, spina ventosa. Notwithstanding this array of causes, the fact that the operation has only once been performed in the large hospitals of this great metropolis for a period of thirty years, affords satisfactory confirmation of our remark, that the cases requiring it are extremely rare. There are enterprising surgeons, who would gladly avail themselves of a fair opportunity of resorting to such an operation, and there are others, whose tried skill and resolution as operators forbid the supposition that they would shrink from undertaking the operation in any case that needed it. The truth is, with active assistants, such as may be obtained at every hospital in the metropolis, the operation, though formidable and dangerous, is not one of any great difficulty.

The author, after making some anatomical remarks on the parts concerned in amputation at the hip-joint, gives a description of the various modes of performing this operation. They are to be found in all recent works on operative surgery, and need not therefore detain us.

The subject of Mr. Cox's operation was Elizabeth Powiss, æt. 23. She had undergone amputation of her left leg above the knee 14 years ago, in consequence of disease of the knee-joint. The integuments around the cicatrix of the stump afterwards ulcerated, and a substance formed on the posterior surface of the stump, which was extremely painful. The ulcerations never entirely cicatrized. About six years after the operation the integuments became hard and thick, and she experienced a sensation as though pins and needles were sticking in the stump; at length, fungous growths showed themselves on the surface, and the parts became more and more painful. She was subjected at different times to a variety of treatment with no permanent benefit. She was re-admitted into the Queen's Hospital, July 1, 1844, at which period the stump presented the following appearances.

"The integuments extending upwards, anteriorly, for about three inches, and posteriorly for about four inches and a half, are of a dull white colour, and of a cartilaginous hardness; and above this, for a limited extent, anteriorly, posteriorly, and laterally, the same parts have a glazed, corrugated appearance, like that presented by an old cicatrix. Patches of fungous growth, of a livid colour, protrude from a half to one-third of an inch from the general surface, at intervals. From these excrescences blood occasionally exudes, and at times a sanious fluid. These excrescences are extremely tender, and bleed on the least touch. The integuments of the upper part of the stump are of a perfectly healthy character and feeling. There is no enlargement of the cutaneous veins, or of the inguinal or femoral glands. The stump generally is tender to the touch; the pain nearly constant, sometimes of a dull, aching character, at other times throbbing." P. 31.

Various remedies were again applied, but to no purpose; and, as the fungoid growths and morbid condition of the integuments continued to extend towards the body, the patient became anxious that amputation should be performed, and the dis-articulation at the hip appeared to Mr. Cox and his colleagues as the only operation likely to be attended with permanent success.

Nov. 1st. The amputation was performed by an anterior and posterior flap. The only circumstance of novelty or interest, which we notice in the account of the operation, is the use of the horse-shoe compressor, invented by Dr. Segnoroni of Padua, which was applied on the external iliac artery as it passes over the body of the os pubis. It completely commanded the hæmorrhage from the anterior flap. It is impossible to follow the author in his tedious and uninteresting narration of the daily and even hourly state of his patient after the operation, and the minute account of the treatment. It is sufficient to mention that her recovery was gradual, and the stump was not entirely healed until Feb. 5th, 1845, more than three months after the operation.

The following account is given of the parts removed.

"*Integuments.*—On making a section of the diseased mass, the whole of the integuments inferiorly to the extent of about four inches were converted into an indurated mass of cartilaginous hardness, of a pearly whiteness, from three-eighths to five-eighths of an inch in thickness. Under the microscope, this structure appeared cellular and vascular, with myriads of minute globules, interspersed with spindle-shaped bodies, as observed by Müller in various tumors.

"*The cellular and adipose tissues.*—These tissues were in considerable quantity; the latter remarkably dense and intersected with fibrous bands.

"*The muscular tissue.*—The muscles presented a peculiar granular appearance, were softened in texture, intersected with fibrous bands, and with the exception of the muscles inserted into the trochanters, appeared to have undergone fatty degeneration." P. 42.

The description is illustrated by a coloured representation of the parts. The disease appears to have been of a fibrous character; and to have formed intractable ulcers, which were morbidly sensitive, the patient being hysterical. We entertain no doubt as to the necessity for the operation in this case. The disease had extended so high up, that Mr. Cox was even compelled to include in the posterior flap a small patch of fungoid growth, which was afterwards destroyed by caustic during the progress of the cure.

At the end of the Memoir we find two statistical tables—one of successful operations—the other, of unsuccessful. During the period of a century there have been, in various parts of the world, 26 successful cases, and 58 unsuccessful cases, making a total of 84. Of these, 29 have occurred in the United Kingdoms, 14 in civil life, and 15 in the army: 8 were successful, and 21 unsuccessful.

We cannot conclude without questioning the good sense and good taste of Mr. Cox in this expensive publication. The Memoir much curtailed—with the omission of the description of the anatomy of the parts concerned in the hip-joint amputation—of the different methods of performing it—and of many particulars in the account of Powiss' case, would have made an excellent paper for the Transactions of a Medical Society, but there is nothing in the Memoir of sufficient interest and importance to render it worthy of separate publication. We think that too much has been made of this case. When we opened the book, and noticed the showy, but not very decent, frontispiece, representing Mr. Cox's patient decked out in a smart cap and gown, &c., displaying her naked abdomen and hip from which the limb had been removed,* it forcibly recalled to our mind some of those street-exhibitions of respectable looking beggars endeavouring to excite the compassion of the charitable, by exposing to view their disgusting deformities. Mr. Cox must know that this coloured plate was not needed to elucidate the case, or to instruct the profession. It is but too obvious, notwithstanding the announcement of the author, which we have quoted in Italics, that this work was intended for the eyes of the non-professional public, rather than for those of professional men, and the impression we received is confirmed by a long list of 262 subscribers, including Prince Albert, of whom 35 only are medical men. Its charitable object is no excuse for the style and mode of this publication, which our respect for the credit and dignity of our profession obliges us to declare is unworthy of a Provincial Hospital Surgeon, a Fellow of the Royal Society, and of the Royal College of Surgeons of England, &c. &c. &c.

---

A Clinical Introduction to the Practice of Auscultation, and other Modes of Physical Diagnosis: intended to simplify the Study of the Diseases of the Lungs and Heart. By *H. M. Hughes*, M.D. Assistant Physician to Guy's Hospital, &c. Small 8vo. pp. 246. Longman and Co. 1845.

The reason assigned by the author for the publication of this manual is that, in his opinion, "a work was wanted in which should be simply explained to the student of auscultation, not merely the origin, character and diagnostic value of certain physical signs, but also the manner in which he should proceed to elicit them." Previous works on the same subject are alleged to be ill-adapted for beginners, in consequence of their containing "such a profusion of terms, and such lots of bruits and râles." We therefore naturally expected to have found that this most vexatious evil was religiously eschewed by Dr. Hughes; but we regret to say that he has not kept to his text in this respect; nor have we been able to discover that his expositions and explanations are at all more simple and intelligible than those given in other treatises upon the subject, or in any of the recently-published Lectures on the Practice of Medicine—Elliotson's or Watson's for example. One striking blemish of his work is the introduction of a good deal of most uncalled-for matter. Among the "Necessary qualifications of the student of Auscultation," we are told that "a certain amount of knowledge of anatomy and physiology is necessary to the practical auscultator," and that "some general knowledge of the ordinary symptoms and results of morbid processes is requisite to the student, at even the very commencement of his

---

* We learn from a note that the profits of the work are to be given to the poor patient.

course." Who could for a moment doubt it? Would any one dream of teaching seamanship to a boy, who had not learned the names of the masts and yards of a vessel? or of instructing him in the use of his quadrant, before he had acquired some knowledge of arithmetic and the elements of astronomy?

The different modes of exploring the chart are treated of under the heads of Inspection or ocular examination—Palpation or manual examination—Percussion—Auscultation—Mensuration—and Succussion.

In the remarks upon the first of these modes, we are informed that it must be "obvious that the surface (of the chest) must be either entirely bare, or clothed with a tight elastic vest." We should think so indeed. Dr. Hughes is also particular in his admonitions to the medical man to be most decorous in all his doings with female patients. Hence, doubtless, the suggestion that, "if the patient be in bed, the lower part of the thorax may be concealed by the bed-clothes, while the superior regions are submitted to inspection, and these in their turn may be covered with a light shawl or handkerchief thrown lightly across the shoulders, while the inferior region is submitted to the same process." In a subsequent part of the volume, the student is told that, "by some persons it may be considered indelicate to apply the naked ear not merely to the bare chest, but even to the unrobed, though slightly covered, parietes of female patients;" and, as if this remark were not sufficient, our fastidious author proceeds to observe that, if it be deemed advisable to apply the ear directly to the chest, "the skin should be covered with some soft yielding material, either appertaining to the patient's dress, or obtained from the attendants." It would be difficult indeed to conceive from what other quarter the doctor is to get the soft material, unless it be out of his own pocket, in the shape of a silk handkerchief. We must not omit to state "that, in the case of uncleanly individuals, some protection should be used;"—moreover, this remark is, by the bye, strictly original, as far as we know—"that, when the chest of male patients is thickly clothed with hair, the hair must be removed before immediate auscultation can be satisfactorily performed!"

In his description of Percussion, Dr. Hughes seems to attach a good deal of importance, in the diagnosis of the early stage of Phthisis, to the character of the sounds elicited by percussing the acromial region (immediately above the clavicles).

At best, the information obtained from percussion in these parts is generally most unsatisfactory, and but little trustworthy. Percussion of the clavicles themselves, and of the infra-clavicular spaces is much more to be depended upon.

In the account that is given of Mediate auscultation, we were a good deal surprised at the following passage. Speaking of the Stethoscope, our author says; this instrument "is merely a conductor. It may, indeed, as in the flexible stethoscope, conduct by the air contained in the hollow of the tube alone; it may, as in the ordinary stethoscope supplied with a nipple-like appendage for the external meatus, conduct both by air within the bore of the cylinder, and by its solid walls,—or it may conduct by the solid walls alone." Are we to understand that the walls o :any sort of stethoscope do not assist in transmitting the sounds from the chest o the patient to the ear of the auscultator?

We had hope dto have been able to have noticed some of Dr. Hughes' observations on the auscultatory phenomena of several pulmonary diseases; but our limited space entirely prevents this. One or two points on cardiac auscultation are all that we can allude to.

The cause of the double sound, or *too-to*, of the heart is thus explained:—

"*Origin of the second Sound.*—If the cock of a leaden pipe, which is not firmly fixed to its solid support, be turned, and a full stream of water be allowed to pass through the aperture which that cock controls, little agitation of the pipe is induced by the mere passage of the fluid. If, however, while the water is passing with rapidity and force, the cock be suddenly turned, and the passage of the fluid through the aperture be in consequence suddenly arrested, a very considerable agitation of the pipe will be immediately perceived, and a corresponding noise will be heard. This agitation, and this noise, are clearly the consequence of the vibrations excited in the fluid by the sudden arrest of its onward progress; which vibrations are communicated to the solid walls of the pipe, and thus become obvious to the eye as well as to the ear. Another illustration may be taken, from what is seen to occur at the closure of a lock on a river. So long as the water continues to flow through the constantly narrowing opening between the two arms of the lock, there is no agitation in the back water. But immediately the two arms meet, a violent shaking is observed in the wood of the lock itself, and in the water which is behind it. This is evidently produced by the sudden stoppage of the stream."

What the turning of the cock does in the one instance, and the closure of the lock in the other, the flapping back of the sigmoid valves is supposed to do in the case of the heart:—"These flood-gates suddenly stop the backward progress of the blood: and this sudden stoppage of the forcible current produces a violent agitation in the particles of the blood, which is communicated to the coats of the vessel, and thence to the parietes of the chest. *Hence arises the second sound of the heart.*"

The *first sound* is, in like manner, produced by the sudden closure of the auriculo-ventricular valves arresting the retrograde progress of the blood into the auricles, during the contraction of the ventricles. "This sudden stoppage of the blood produces a violent agitation in the fluid, which is communicated to the body of the ventricles, and thence to the parietes of the chest."

With respect to the differences in the exact situation or point of the chest, at which abnormal sounds or murmurs are heard, and in the exact moment of time, at which they occur, viewed as characteristic indications of what particular valves are mainly affected, the impression, left on the reader's mind by Dr. Hughes' description, will (we should say) be that the diagnosis of the precise seat of the cardiac disease is considerably easier than he will really find to be the case in practice. The discordance of opinion among recent writers on these very points sufficiently attests how difficult this discrimination is. As an example of the very hypothetical character of many of the statements on the subject of cardiac auscultation, we may adduce the following short paragraph as to the supposed seat of the blowing and other murmurs, that are so commonly heard over the region of the heart in chlorotic girls. "They are very generally supposed to be confined to the aortic openings. This is certainly a mistake. They are most assuredly very frequently connected with the pulmonary artery, in which murmurs, quite independent of any disease of the vessel, or of its valves, are far from uncommon."

What, pray, are the grounds for this *assured* belief? And here we may, *en passant*, allude to another point in the history of Chlorotic murmurs, wherein most sage doctors differ. In Dr. Hughes' description of the "continual humming sound" or "venous murmur," so frequently met with in the necks of chlorotic girls, we read that "it most probably depends upon partial obstruction to the quickened flow of blood through the veins. Strong pressure causes it to cease; but without pressure, *directly or indirectly applied*, it is, I believe, never heard." Compare this statement with that of Dr. Latham in his recent volume, of which we gave so ample a review in the number of this Journal for last July: "The truth is, a very free current of blood is essential to the production of this venous murmur. A slight degree of pressure upon the vein will alter its character, and pressure, very far short of that which would arrest the current of the blood, will abolish it altogether." This very peculiar sound is sometimes remarkably distinct in that triangular space between the outer edge of the sterno-mastoid muscle and the clavicle: it conveys the idea of a continuous rushing of a fluid along a tube, and is not unlike that of the water rushing up the main pipe of a house, when the water is "turned on."

If there is one reflection more than another that has been forced upon our minds by the perusal of Dr. Hughes' work, it is that of the danger of trusting too much to what are called the *physical*, and too little to what have been (absurdly enough) denominated the *rational* symptoms, in our diagnosis of pulmonary and cardiac diseases. Far be it from us to undervalue the importance of Auscultation; it is unquestionably a most useful handmaid or auxiliary, but *by itself* it is apt to become a most arbitrary mistress, and a most deceptive guide.

---

### MEDICAL REFORM.

An Address (The Third). By the Society of Apothecaries. Highley, 1845.

Le Journal Officiel du Congres Medicale de France. Paris, 1845.

As we foresaw, the apathy of a portion of the general practitioners, and the violent dissension of another portion, have encouraged the two incorporated bodies to oppose their just demands, and indefinitely delayed the accomplishment of their wishes. We say indefinitely, for he must be a bold man, who, in the present state of politics, can imagine a Home-secretary, with sufficient leisure and inclination to examine into the contradictory statements with which he is supplied. Who knows, too, but some other minister may require as laborious a process of conversion or education bestowed upon him as did Sir James Graham. Under the present state of things then we need not enter into any examination of the last Bill, as, like its predecessors, it is doubtless destined to undergo alterations before being presented to Parliament. In the mean time, we may observe that we entirely agree in the opinions put forth in the "Address," and in the "Transactions" of the National Association, viz. that the arrangement entered into with Sir James Graham was satisfactory, and should be considered final, as far as concessions on the part of the general practitioner are concerned, and that no Bill should be received unless harmonizing with such arrangement. In the present uncertain state of affairs, the only means the profession has for obtaining its rights is the maintenance of the firm front which the union of numbers alone can give, and therefore we consider the continuance of the organization of the "National Association" is of the last importance. It is the most imposing body ever yet marshalled in the cause of Medical Reform, and by the temperate, yet firm, line of conduct it has pursued, has well earned the confidence of the profession. The willingness, with which the Apothecaries' Company has offered to divest itself of its present privileged position, is highly praiseworthy. Looking upon the efficient examination of future practitioners as by far the most essential of all the circumstances to be provided for, we cannot but rejoice that this is not to be undertaken, as at present, by his own grade of the profession alone. This is the leading defect in the present system, and whether it is to be remedied by the more simple plan proposed by the Association of selecting eminent men to aid the examiners, or by the more complex system of a double examination, is of comparatively little importance.

The extreme section of medical reformers in this country have been always accustomed to cite the condition of the Profession in France as one especially serviceable, and infinitely superior to that which prevails among ourselves. Strange to say, our neighbours have long been of a very different opinion, and have demanded and been promised a reformation respecting it for the last twenty years. What the government has failed to produce, a voluntary association meeting at Paris in November, the "*Medical Congress*," has endeavoured to originate. Although subsequent to our own movements, that in France was not undertaken in imitation of it, but more from an almost random suggestion of M. Amedée Latour, the talented and witty contributor to the *Gazette des Hôpitaux*, under the pseudonym of "*Jean Raimond*." The rapidity with which this was acted upon proves a mine of discontent was already laid, upon which it operated as the spark. Five thousand names were enrolled, and from 600 to 1000 persons assisted at the deliberations of the Congress during its fortnight sitting at Paris. The business was well, systematically, and almost unanimously executed, consisting chiefly of detailed and elaborate expositions of the present position of the profession in France, and of the urgent necessity for its amendment. Although the Congress was regarded as a hostile demonstration by most of the professors and hospital officers of Paris, yet several men well known to the profession took part in its deliberations, and the Minister himself was present on more than one occasion. The Crown has already nominated a Commission for inquiry into the state of medical education, and has promised, ere long, to prepare a Bill, embodying at least some of the proposals of the Congress, though by no means giving sanction to others. Having perused the debates of the Congress with great care, we have come to the conclusion, that the practical evils, under which the profession labours in France, are far greater than those which have afflicted it in our own country. We can, however, only glance at a few of these, in noticing some of the conclusions arrived at by the Congress.

1. That there should be but one order of medical practitioners. This is not so visionary a demand in France as it would prove in our own country. Under the title of *Officiers de Santé*, half-educated practitioners are allowed to practice, and are distributed over the whole of France, to the amount of some 8000 to the 19 or 20,000, or according to others 28,000, doctors of medicine or surgery. These men are furnished with the merest rudiments of knowledge, and yet among the ignorant frequently contrive to oust their better-educated neighbours. A man, who is rejected as too ignorant for a doctor's degree, may at once obtain a right to practice as an *Officier de Santé*. The number of these men, compared with that of other practitioners, is upon the increase, and so great is the evil done to the profession by the admission of such a class, that its suppression is everywhere demanded. This is not so easily effected; for ignorant as they are, for the most part, they seem to have possessed themselves of considerable influence, while their suppression would leave the poor and thinly-habited districts of France unprovided with medical aid. It seems, however, that in rural districts the profession has not only, or even chiefly, to struggle against these men, but against the *clergy*, who impudently take on medical pretensions, and do not hesitate even to pass pessaries, or attend women in child-birth!

2. That although fees in private practice should not be regulated by law, yet when a practitioner is called upon public services, he should be liberally dealt with. At present it is quite the reverse of this; for 2 francs seems to be the sum which practitioners get for the same services in law-courts, &c. for which ours receive their guinea.

3. The right of claim of a debt for attendance shall extend to five years. At present the debt is not recoverable if not applied for within the year after treatment has commenced.

4. A more efficient repression of illegal practitioners. However peremptory the laws against quackery may seem to be, they are rendered inefficient by the power of enforcing penalties being optional, and by the ridiculous small amounts to which these extend. We gather from the accounts of the different speakers, that illegal practice is as rife in France as with us. Quack advertisements equally infest the public places and journals of Paris as of London; and worse still, we find the columns of the *Gazette des Hôpiteaux*, the stern denouncer of all abuses, admitting announcements of the widely celebrated "Pilules Morison," and the like.

5. Foreign practitioners settling in France are to undergo the same examinations and give proof of the same courses having been attended as French students do.* One can hardly believe that so unjust and insulting a proposal could have been made and carried. According to this, no matter what eminence a man may have obtained, or what diploma in proof of this he may present, he is to undergo the same ordeal as if he had never been examined or studied at all.

6. The midwives form a very large body in France, very much greater in proportion than with us. The Congress does not venture to ask for their suppression, although not doubting the benefit of their abolition, if their place could be supplied; but insists that they shall be prohibited undertaking any operations but bleeding and vaccination.

7. The Congress demands that all appointments to hospitals, &c. shall be by *concours*. This is by no means the case at present, some only of these being so filled up. We certainly deem this the least objectionable mode, and many of the most eminent men in France would never have been heard of but for its operation. It is, however, anything but the pure process at all times which some believe, since the judges are not infrequently canvassed and endeavoured to be influenced by all sorts of underhand procedures. The Congress also considers it very unjust, that after a man has qualified himself for the post of hospital physician, &c. and has obtained it, he is yet liable to removal every five years by the council or minister. It insists likewise that Professors reaching the age of sixty-five should fall back into honourable and easy retirement.

We will only notice two other circumstances connected with the deliberations of this body: one is the revolting fact, that, whenever any mention is made of providing the necessaries for instruction in the schools, "live animals for operations and experiments" are mentioned as a matter of course. M. Amussat says they form "the natural transition between operations upon the subject and upon man!"

The other circumstance is that the *pharmaciens* (chemists with prohibition from prescribing and monopoly in compounding) and veterinary surgeons, furnished considerable contingents; and that the former set their faces steadily against quack nostrums and advertisements, illegal practice of medicine, and connivance between chemists and practitioners, common in France as well as here.

It will be seen then, that the profession in France has many subjects of complaint in common with ourselves, and some we are happily free from. At all events, it is at present no model for our imitation.

---

* During the discussion upon this topic, M. *Malgaigne*, one of the ablest surgeons of France, and the popular orator of the Congress, displayed the grossest ignorance of the nature of the qualifications of the practitioners in England, as the following passage shews: "They tell you that foreign universities are as good as our own, and they have spoken of England. There, there are no Faculties, but only Private Schools which send forth three classes of practitioners. First there are the *physicians*, a grade corresponding to that of our doctor of medicine, and who are truly learned and able men; then come the *surgeons*, our *officiers de santé*; and lastly, what they call the *apothecaries*, whom I can only compare with the *barbers* of the eighteenth century. Well, are these men, these barbers, to come over here and demand to be assimilated with our doctors? That can never be!"

The Descriptive and Physiological Anatomy of the Brain, Spinal Cord, and Ganglions, and of their Coverings. Adapted for the Use of Students. By *Robert Bently Todd,* M.D. F.R.S. 8vo, pp. 284. London: 1845.

We are happy to find that Dr. Todd has published, in a separate and convenient form, his account of the Nervous Centres, which appeared originally in the Cyclopædia of Anatomy and Physiology. Dr. Todd states that he "has been induced to submit this book to the public in its present shape, by the frequent complaints which have reached him from students and others, of the want of a description of the brain and spinal cord, embodying the most recent observations on the anatomy of these important organs." We have, in the two preceding numbers of this Journal, had occasion to speak in such favourable terms of the article "Nervous Centres," that it only remains for us to state, in this place, that the work before us being, as the author informs us, a reprint of this article, will place those who study it on a level with the existing knowledge in this interesting department of animal organization. The treatise is illustrated with a considerable number of well-executed and judiciously-selected wood-cuts; and we can, with strict justice, unhesitatingly recommend it, not only to those for whose use the author has more particularly designed it, but also to those who are engaged in active practice.

---

Leçons d'Anatomie Comparée de Georges Cuvier. 2de Edition, corrigée et augmentée. Tome Troisième; contenant le Système Nerveux et les Organes des Sens. revu par MM. *F. G. Cuvier* et *Laurillard.* Paris, 1845.

It is well known that the illustrious author of the *Leçons d'Anatomie Comparée* had, for a long series of years preceding his lamented death, devoted his great talents and unceasing industry to the preparation of what he called his "Great Comparative Anatomy," which, if he had been spared to realise the conception, would have been to these "Leçons," what the *Regne Animal* was to its humbler predecessor, the "Tableau élémentaire des Animaux"—the finished picture as contrasted with the mere outline.

The Editors state, in their Preface to the first volume, that Cuvier, "during thirty years, had never ceased accumulating, in his collection and in his portfolios, the materials for this immense undertaking." But the magnitude of the enterprize and the consequently distant period of its achievement, induced him to render these "lectures," in the interval, as complete as possible: in this way arose the idea of this augmented and corrected second edition, in the preparation of which he was occupied with his accustomed ardour when he was, alas! surprized by death. There attaches thus a peculiar interest to these tomes, bearing, as they do, the last impress of genius, and, imperfect as in some respects they are, they will be treasured, in after-times, like the unfinished sketches of the great masters of painting, Raffaelle, Domenichino, and the Caracci.

"In the volume lately published, the Editors inform us, it has been their aim to present, in the limited space of a volume, a résumé of the immense number of works of which the nervous system and the organs of the senses have been the object during thirty years; and at the same time to preserve, as far as that was possible, the frame-work and the text of the first edition;" a task of great difficulty, especially as it appears that this portion of the lectures had not received the advantage of Cuvier's own revision. By availing themselves of the many and important additions made to this branch of Comparative Anatomy by judicious critiques, MM. F. Cuvier and Laurillard have succeeded in presenting a comprehensive view of the existing state of knowledge. We do not, however, propose to enter into any of the descriptive details, which are evidently more calculated for the guidance of the student in the cultivation of this delightful science, than for the pages of a review; the extended notice of the Nervous System, contained in the two preceding numbers of this Journal, would moreover render any additional notice misplaced.

We shall, for these reasons, merely state that, as the new edition of the Anatomie Comparée is now all but completed, one volume only remaining to be published, those of our readers, who are interested in these inquiries, will do well to procure this classical work of one of the most illustrious naturalists of modern times.

---

Outlines of Chemistry. By *William Gregory,* M.D., Professor of Chemistry in the University of Edinburgh, &c. 12mo, pp. 588. London, Taylor and Walton. 1845.

These Outlines will be a most useful text-book to the student, while attending a course of lectures on Chemistry. They contain an immense amount of information in as small a bulk as possible, and the high character of the Professor will be a sufficient voucher for its general accuracy—as far, at least, as accuracy can be looked for in the details of a science, which "is, at present, not only in a state of rapid development and progress, but in a state of transition." Every year is adding not only a host of new facts, but suggesting also a multitude of new views, in Chemistry. The great difficulty, which the general reader must experience in keeping up with the progress of this most interesting branch of knowledge, is the enormous multiplication of new terms.* One advantage indeed of this must be to force the student to have frequent recourse to his Greek and Latin Lexicons. We may mention that Dr. Gregory has entirely excluded from these Outlines any description of what are usually called the "Imponderables"—Light, Heat, Electricity,and Magnetism:—regarding them to belong to the domain rather of Physics than of Chemistry. The first part, occupying 236 pages, is devoted to the consideration of *inorganic,* and the second part, which contains upwards of 300 pages, to that of *organic* substances. As a matter of course, all the most recent hypotheses as to the constitution of Acids and Salts, the existence of organic radicals, and so forth, are minutely described and canvassed. The parts may be purchased separately.

---

* Dr. Prout, after enumerating the following "extraordinary names"—*Alantoin, Cyanuric acid, Cyanelid, Alloxanic acid, Mesoxalic acid, Mykomelinic acid, Parabanic acid, Uramil,* &c.—of various alleged products of urea and uric acid, says: "I must protest against the barbarism of the terms; particularly as I am by no means satisfied that the doctrines, on which they are founded, are satisfactorily established."

An Essay on the Use of Narcotics, and other Remedial Agents, calculated to produce Sleep, in the Treatment of Insanity. By *Joseph Williams,* M.D. Pp. 120. London, Churchill. 1845.

This Essay presents a succinct and very neat exposition of the various remedies that have been recommended for the purpose of soothing the excessive irritability and sleeplessness in the different forms of Insanity. Bleeding—purgatives—emetics—narcotics—the warm-bath, &c., are severally discussed, and the value of each is very fairly and judiciously stated. On the much vexed question as to the exhibition of opium in Insanity, Dr. Williams shews his good sense by distinguishing the cases where it is advisable, from those where its use is questionable, if not positively injurious. He has omitted to allude to one indication for its employment, which we regard to be of decided practical value, and to which we would therefore invite his attention, and that of our readers generally. This is the state of the urine. Whenever this secretion is high-coloured and scanty, let opium be eschewed in every form, until the bowels and kidneys, at least, have been freely relieved; should the symptoms not call for more active depletion, by the abstraction of blood. Even when the urine is less decidedly affected, the combination of alkaline salts with sedatives will often be found most beneficial. The more we see of practice, the more importance we are inclined to attach to the frequent examination of the renal excretion, more especially in cases of nervous disturbance.

---

